# Kosten-Erlös-Defizit der ambulanten Versorgung von Bagatellverletzungen in der Notaufnahme

**DOI:** 10.1007/s00113-022-01205-9

**Published:** 2022-07-07

**Authors:** Nils Mühlenfeld, Jan Tilmann Vollrath, Jason-Alexander Hörauf, Oliver Schöffski, Jasmina Sterz, Julia Riemenschneider, Philipp Störmann, Ingo Marzi, René D. Verboket

**Affiliations:** 1 Klinik für Unfall‑, Hand- und Wiederherstellungschirurgie, Goethe Universität Frankfurt, Theodor-Stern-Kai 7, 60590 Frankfurt am Main, Deutschland; 2 Lehrstuhl für Gesundheitsmanagement, Friedrich-Alexander-Universität Erlangen-Nürnberg, Nürnberg, Deutschland

**Keywords:** Selbstvorstellung, Kapazitätsdefizit, Notfallversorgung, Gesundheitsökonomie, Self-introduction, Capacity deficit, Emergency care, Health economics

## Abstract

**Hintergrund:**

Viele Patienten mit Bagatellverletzungen gehen heutzutage häufig vorschnell in die Notaufnahmen und binden dort Ressourcen und Personal.

**Ziel der Arbeit:**

Das Erstellen des Kosten-Erlös-Verhältnis der ambulanten Versorgung von Bagatellverletzungen in der unfallchirurgischen Notaufnahme.

**Material und Methoden:**

Die Kalkulation erfolgte anhand der einheitlich abgerechneten Notfallpauschalen des Einheitlichen Bemessungsmaßstabes (EBM). Mittels der gängigen Tarifverträge für Ärzte und Pflegepersonal wurden Minutenkosten berechnet. Der zeitliche Behandlungsaufwand wurde anhand von 100 Referenzpatienten mit einer Bagatellverletzung ermittelt. Die Fallkostenkalkulation mit den jeweilig anfallenden Ressourcen erfolgte mit dem operativen Controlling des Universitätsklinikums Frankfurt.

**Ergebnisse:**

Eingeschlossen wurden 4088 Patienten mit Bagatellverletzungen, welche sich 2019 eigenständig fußläufig vorstellten. Die häufigsten Gründe für die Vorstellung waren Prellungen der unteren (31,9 %; *n* = 1303) und oberen Extremität (16,6 %; *n* = 677). Kalkuliert wurden Zeitaufwände von 166*,*7 min/Tag für das ärztliche und 213,8 min/Tag für das Pflegepersonal. Es wurde ein Gesamterlös von 29.384,31 € und Gesamtlosten von 69*.*591*,*22 € berechnet. Somit lässt sich ein Erlösdefizit von 40.206,91 € für das Jahr 2019 berechnen. Das entspricht einem monetären Defizit von 9,84 €/Patienten.

**Diskussion:**

Es herrscht Knappheit an der medizinischen Ressource „Personal“, um das heutzutage hohe Aufkommen an sich selbst vorstellenden fußläufigen Patienten mit Bagatellverletzungen zufriedenstellend und ökonomisch zu bewältigen. Die bisherige Vergütung der Behandlung von Bagatellverletzungen durch den EBM ist für den Krankenhaussektor unzureichend.

## Abstract (en)

**Background:**

Many patients with minor injuries hastily present in the emergency department and tie up resources and personnel there.

**Objective:**

To establish the cost-revenue relationship of outpatient care of minor injuries in the traumatology emergency department.

**Material and methods:**

The calculation was based on the uniformly billed emergency flat rates of the uniform assessment standard (EBM). Using the current collective bargaining agreements for physicians and nurses, per minute costs were calculated. The time required for treatment was determined on the basis of 100 reference patients with minor injuries. The case cost calculation with the respective resources was carried out with the operational controlling of the University Hospital Frankfurt.

**Results:**

A total of 4088 patients with minor injuries who presented in 2019 were included. Most common reasons for presentation were contusions of the lower (31.9%; *n* = 1303) and upper extremities (16.6%; *n* = 677). A time expenditure of 166.7 min per day for the medical staff and 213.8 min per day for nursing staff was calculated. A total revenue of 29,384.31 € and total costs of 69,591.22 € were calculated. Thus, a revenue deficit of −40,206.91 € can be calculated for the year 2019. This corresponds to a monetary deficit of 9.84 € per patient.

**Conclusion:**

There is a shortage of the medical resource “personnel” to satisfactorily and economically manage the nowadays high volume of self-presenting pedestrian patients with minor injuries. The current remuneration of the treatment of minor injuries by the uniform assessment scale is insufficient for the hospital sector.

**Graphic abstract:**

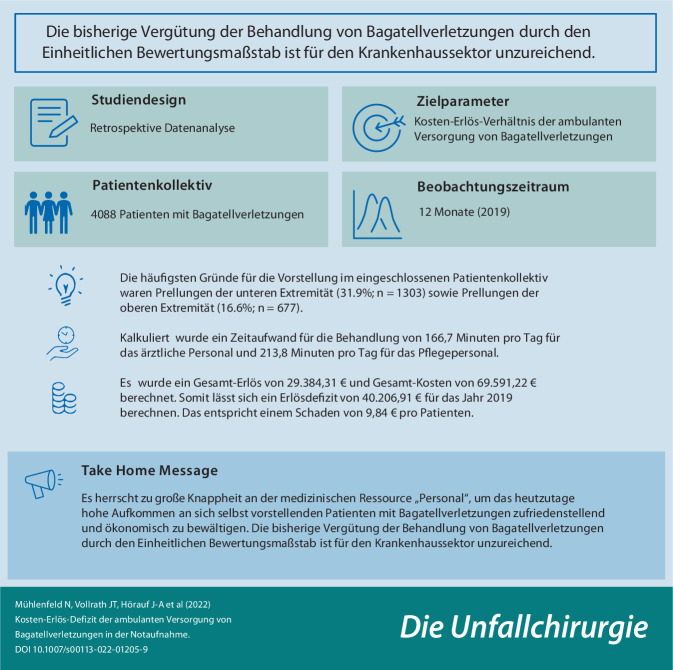

## Hintergrund und Fragestellung

Die Akutbehandlung verletzter Patienten ist nicht nur eine humanitäre und gesellschaftliche, sondern auch eine volkswirtschaftliche Aufgabe [7]. Jährlich werden nahezu 18 Mrd. € (4,8 % der gesamten Ausgaben im Gesundheitswesen) für die Behandlung von Verletzungen, Vergiftungen und bestimmter anderer Folgen äußerer Ursachen ausgegeben (Stand 2016) [26].

Überfüllte Notaufnahmen und Personalmangel sind heutzutage Realität in den meisten Krankenhäusern Deutschlands. Dabei zeigte sich in den letzten Jahren ein kontinuierliches Ansteigen der eigenständigen, durch die Patienten initiierten Vorstellungen in der Notaufnahme [1, 9, 15, 21]. Knapp über die Hälfte (52 %) der 2 Mio. Behandlungen in den Notaufnahmen der öffentlichen Krankenhäuser erfolgen ambulant. Die Abrechnung der Mehrheit der Fälle (80 %) erfolgt mittels der gesetzlichen Krankenversicherung (GKV) über den einheitlichen Bewertungsmaßstab (EBM) [23].

Die Versorgung von Bagatellverletzungen im Rahmen der unfallchirurgischen Notfallversorgung ist fester Bestandteil des klinischen Alltags. Bagatellverletzungen sind Verletzungen, die keine akute Behandlung durch einen Notarzt oder ein Krankenhaus erfordern. Ihre Behandlung stellt auch für unerfahrene Chirurgen eine eher geringe technische Herausforderung dar. Allerdings entsteht nicht selten ein Personal- und Zeitproblem, da ein Team aus einem Chirurgen und einer Pflegekraft gleichzeitig beim Patienten gebunden wird. Diese Problematik wird zusätzlich durch den in Krankenhäusern herrschenden Facharztstandard verstärkt, da Befunde und Behandlungen mit einem Facharzt rückgesprochen werden müssen. Zusätzlich wird vermutet, dass Bagatellverletzungen durch die oft unzureichende Vergütung nach dem EBM zu einer regelhaften Kostenunterdeckung in den Notaufnahmen beitragen [9, 17, 20]. Es ist allerdings bislang noch nicht genau untersucht, in welchem Ausmaß dies der Fall ist.

Der ärztliche Bereitschaftsdienst (ÄBD) soll diejenigen Patienten notfallmäßig behandeln, deren Erkrankungen, Vergiftungen und Verletzungen keinen Aufschub bis zur regulären Sprechstunde (meistens der nächste Werktag) der niedergelassenen Ärzte erlauben, jedoch keinen Rettungsdienst oder eine sofortige Notfallbehandlung in einem Krankenhaus erfordern und somit eine Entlastung der überfüllten Notaufnahmen bewirken. Dennoch zeigte sich zwischen 2009 und 2015 ein Rückgang der Notfälle um 15 %, welche durch den ÄBD behandelt wurden. Gleichzeitig stieg jedoch die Anzahl der Notfälle um 42 %, von 6 Mio. von 2009 auf über 8,5 Mio. Fälle im Jahr 2015. Somit ist eine deutliche Verschiebung der Notfallvorstellung in die Notaufnahme erfolgt [15, 20].

Ziel dieser Arbeit ist es, das Kosten-Erlös-Verhältnis der ambulanten Versorgung von Bagatellverletzungen in der unfallchirurgischen Notaufnahme zu erstellen. Somit soll der gesundheitsökonomische Mehrkostenaufwand, der aufgrund von Bagatellverletzungen für das Universitätsklinikum Frankfurt am Main (anhand des Beispieljahres 2019) entstanden ist, ermittelt werden. Eingeschlossen wurden alle gesetzlich versicherten Patienten, welche sich 2019 selbstständig in der unfallchirurgischen Notaufnahme des Universitätsklinikums Frankfurt am Main vorstellten, jedoch auch durch den ambulanten Sektor (Hausärzte, ÄBD) hätten behandelt werden können.

## Studiendesign und Untersuchungsmethoden

### Design und Datenerhebung

Die vorliegende Studie erfolgte unter Zustimmung der Ethikkommission der medizinischen Fakultät der Goethe Universität Frankfurt am Main (Ethik-Votum: **2021-380**). Sie folgt den STROBE-Richtlinien für Beobachtungsstudien (Strengthening The Reporting of Observational Studies in Epidemiology) sowie den RECORD-Richtlinien für Observationsstudien (Reporting of studies Conducted using Observational Routinely-collected Data) [10, 24]. Es erfolgte die retrospektive systematische Auswertung einer aufeinanderfolgenden Kohorte von Patienten, welche mit einer Bagatellverletzung in der zentralen Notaufnahme des Universitätsklinikums Frankfurt am Main vom 01.01.2019 bis zum 31.12.2019 vorstellig wurden.

In dieser Studie werden Verletzungen als Bagatellverletzungen zusammengefasst, für die keine umfangreiche chirurgische Behandlung notwendig war und somit durch den Hausarzt oder den niedergelassenen Kollegen in der normalen Sprechstunde sowie durch den ärztlichen Bereitschaftsdienst in der Notfallversorgung hätte erfolgen können. Darin eingeschlossen werden Prellungen, kleinere Schnittwunden, kleinere Verbrennungen und Verbrühungen bei Erwachsenen von unter einem Prozent der Körperoberfläche fernab von Gelenken bis zum Grad 2A, Distorsionen der Halswirbelsäule sowie kleinere Abszesse und Panaritien, bei denen keine komplexe chirurgische Behandlung durchgeführt werden muss. Eingeschlossen werden ausschließlich Patienten, welche sich fußläufig und selbstständig in der zentralen Notaufnahme vorstellten. Patienten mit Bagatellverletzungen aufgrund eines potenziell gefährdenden Traumamechanismus (Hochrasanztraumata, Sturz aus mehr als 3 m Höhe, Schnittwunden an potenziell lebensbedrohlichen Körperstellen o. Ä.) werden nicht zum Patientenkollektiv gezählt und somit von den Ergebnissen dieser Studie ausgeschlossen.

### Kosten-Erlös-Kalkulation

Die Kalkulation der Erlöse in dieser Arbeit erfolgte anhand der einheitlich abgerechneten Notfallpauschalen EBM 01205 und EBM 01207 [13]. Die Wundversorgungen wurde im Falle kleiner Wundversorgungen mit weniger als 5 min Zeitaufwand mit EBM 02300 und bei größerem Aufwand mittels EBM 02301 abgerechnet [14]. Die Abrechnung eines stabilisierenden Verbandes erfolgte mit der Pauschale EBM 02350 ([27]; Tab. 1).

**Table 1:** 

EBM	Beschreibung	Vergütung
**01205**	Bagatellerkrankung/Verletzung *Montag bis Freitag 7:00–19:00 Uhr*	4,79 €
**01207**	Bagatellerkrankung/Verletzung *Montag bis Freitag 19:00–7:00 Uhr, Samstag/Sonntag/Feiertag*	8,52 €
**02300**	Wundversorgung (5 min Dauer)	6,07 €
**02301**	Kleinchirurgischer Eingriff II *Abszesseröffnung oder Fremdkörperentfernung*	13,74 €
**02350**	Anlegen stabilisierender Verband	11,29 €

Die Fallkostenkalkulation wurde in Zusammenarbeit mit dem operativen Controlling des Universitätsklinikums Frankfurt erstellt. Diese erfolgte analog zu einer vorangegangenen Arbeit des Autors dieser Arbeit („Kosten-Erlös-Defizit der ambulanten Versorgung von Kopfplatzwunden in der Notaufnahme“, veröffentlicht in *Der Chirurg *[17]). Die anfallenden Einzelkosten der Ressourcen sind in den Tab. 3 und 4 aufgeführt und die Aufschlüsselung der jeweilig anfallenden Kosten dargestellt. In Bezug auf die Personalkosten wurden anfallende Kosten für Ärzte und das Pflegepersonal getrennt betrachtet. Zulagen, Überstunden oder andere Formen von Zuschlägen wurden nicht miteinbezogen.

Grundlage für die Minutenkosten des ärztlichen Personals in der Notaufnahme ist die seit dem 20.02.2019 geltende Entgelttabelle nach Tarifvertrag für Ärzte an den hessischen Universitätskliniken (TV-Ärzte Hessen 2018) [16]. Um einen repräsentativen Assistenzarzt in der Notaufnahme zu evaluieren, wurde das Durchschnittsgehalt für Assistenzärzte aus dem ersten, dem vierten und dem siebten Weiterbildungsjahr berechnet. Es wurden bei einer 42-h-Woche im Notaufnahmedienst die Minutenentgelte sowie der Arbeitgeberanteil für die Lohnnebenkosten im Jahr 2019 berechnet. Dafür wurden Renten‑, Arbeitslosen‑, Kranken- und Pflegeversicherung für einen imaginären Arbeitnehmer mit Lohnsteuerklasse 1, gesetzlicher Krankenversicherung und ohne Kinder als Lohnnebenkosten berechnet. Unter der Berücksichtigung einer Ausfallzeit von 2,1 % wurden die Nettojahresarbeitsstunden kalkuliert [11]. Grundlage für die Minutenkosten des Pflegepersonals in der Notaufnahme ist der Tarifvertrag der Johann-Wolfgang-Goethe-Universität Frankfurt am Main (TV-G-U) in der Fassung des Änderungstarifvertrags vom 11.09.2017. Hierfür verwendet wurde die Entgelttabelle für Pflegekräfte, welche am 01.01.2018 in Kraft trat. Dabei erfolgte die Kalkulation aufgrund der vertraglich vereinbarten 38,5 Wochenstunden für eine Referenz des Pflegepersonals aus der vorwiegend beteiligten Entgeltgruppe P8. Anhand der durchschnittlichen Personalkosten für die Stufen 3 und 4 wurde der Arbeitgeberanteil der Lohnnebenkosten entsprechend berechnet. Dies erfolgte unter Berücksichtigung der durchschnittlichen Ausfallzeit von 6,85 % [11]. Zusammen mit der Klinikapotheke und dem Einkauf der Universitätsklinik Frankfurt wurden die Kosten für Verbrauchsmaterial, Desinfektionsmittel und Lokalanästhetikum, stützendes Verbandsmaterial und weiteres Nötige erhoben. Im Falle notwendiger stabilisierenden Verbände wurden zur Vereinfachung die durchschnittliche Materialmenge und Materialkosten aus einer Unterschenkelgipsschiene, einer Unterarmgipsschiene und einer Oberarmgipsschiene angegeben. Anfallende Leistungen der Radiologie wurden nicht berücksichtigt. Die Kosten der somit ermittelten einzelnen Faktoren wurden miteinander addiert. Der sich daraus ergebende Gesamtkostenbetrag wurde mit der Gesamtvergütung nach EBM verglichen.

### Kalkulation des Zeitaufwandes der Behandlung

Der zeitliche Behandlungsaufwand durch das ärztliche zusammen mit dem pflegerischen Personal wurde anhand 100 Referenzpatienten mit einer Bagatellverletzung dokumentiert und daraus der durchschnittliche Behandlungsaufwand gebildet. Die Dauer des Erstkontaktes und der initialen Untersuchung wurde dabei mit dem Zeitaufwand für das Erstellen des Ambulanzbriefes und der Dauer des Abschlusskontakts addiert. Hierzu wurden die Besprechung der ggf. erhobenen radiologischen Befunde sowie die Erklärung des Therapieprozedere mit ggf. anschließend anfallender Anlage eines stabilisierenden Verbandes und der Aushändigung von Rezepten gezählt. Im Falle kleinerer Wunderversorgungen sowie der unkomplizierten Spaltung von kleineren Panaritien und Abszessen wurde ein zusätzlicher Durchschnittsminutenaufwand von 5 min entsprechend einer Standardwundversorgung mittels einschichtiger Hautnaht zugrunde gelegt.

### Statistische Auswertung

Die erhobenen Daten wurden mittels deskriptiver Statistik analysiert. Hierzu wurden zunächst alle Daten in ein Tabellenprogramm eingegeben (Microsoft Excel, Redmont/WA, USA). Es erfolgte die Auswertung der absoluten Patientenzahl anhand ihrer Diagnose, ihrer Abrechnungsziffer nach EBM sowie ihres prozentualen Anteils an dem untersuchten Gesamtkollektiv. Die statistische Auswertung erfolgte mittels Graphad Prism 9 (San Diego, Kalifornien, USA).

## Ergebnisse

### Patientenzahlen und Art der Vorstellung

Nach Auswertung der digitalen Datenbanken konnten insgesamt 9129 Patienten mit unfallchirurgischen Diagnosen identifiziert werden, die über die zentrale Notaufnahme des Universitätsklinikums Frankfurt am Main vorstellig wurden (Einweisung durch den ÄBD: 4,0 %; *n* = 367; Notfalleinweisung durch den Hausarzt: 7,6 %, *n* = 692). Nach Auswertung der Entlassdiagnosen verblieben 4257 Patienten mit Bagatellverletzungen. Von diesen wurden 96,0 % (*n* = 4088) aus eigener Motivation und ohne vorangehende Sichtung oder Einweisung durch den ÄBD oder den Hausarzt vorstellig. Einweisungen durch den ÄBD erfolgten in 2,9 % (*n* = 125), durch den Hausarzt in 1,0 % (*n* = 44) der Fälle. Somit verblieben in der finalen Auswertung 4088 unfallchirurgisch behandelte Patienten mit Bagatellverletzungen, welche sich in 2019 fußläufig in der zentralen Notaufnahme vorgestellt hatten. Das entspricht einem Anteil von 44,8 % aller unfallchirurgischer Fälle.

Die meisten sich fußläufig vorstellenden Patienten mit Bagatellverletzungen suchten die Klinik am Freitag (*n* = 706; 17,3 %), am Dienstag (*n* = 657; 16,1 %) und am Sonntag (*n* = 589; 14,4 %) auf. Die Verteilung der Patienten anhand des Zeitpunktes ihrer Vorstellung zeigt eine gehäuftes Patientenaufkommen nachmittags gegen 16:00 Uhr (*n* = 461; 11,3 %) sowie vormittags gegen 9:00 Uhr (*n* = 413; 10,1 %).

### Diagnose und Therapie

Der häufigsten Gründe für die Vorstellung im eingeschlossenen Patientenkollektiv waren Prellungen der unteren (*n* = 1303; 31,9 %) sowie der oberen Extremität (*n* = 677; 16,6 %). Vorstellungen aufgrund von chronischen Beschwerden waren hauptsächlich Arthrosebeschwerden (*n* = 218; 5,3 %) und Tendovaginitiden im Bereich des Handgelenks (*n* = 98; 2,4 %). Vorstellungen aufgrund einer kleineren Wunde oder eines kleineren Abszesses/Panaritium erfolgten in insgesamt 3,5 % (*n* = 145) der Fälle.

Nach der ggf. notwendigen radiologischen Diagnostik zum Ausschluss von Frakturen wurden bei 143 der 1980 Patienten mit Prellungen stützende Verbände angelegt (7,2 %). Die chirurgische Spaltung von kleineren unkomplizierten Abszessen/Panaritien wurde in 62,5 % (*n* = 30 von 48) erforderlich. Die Therapie mittels chirurgischer Naht erfolgte bei 47 von 97 Patienten mit kleineren Wunden (48,5 %).

### Kalkulation des Zeitaufwandes der Behandlung

Für das Aufnahme- und Abschlussgespräch zusammengerechnet mit dem Erstellen des Arztbriefes und ggf. anfallenden Rezepten oder Arbeitsunfähigkeitsbescheinigungen durch den Arzt wurde eine durchschnittliche Dauer von 14,8 ± 3 min (95 %-KI = 14,2–15,5) dokumentiert. Dabei wurden sowohl die Arztbriefe als auch die ggf. anfallenden Rezepte durch den Arzt selbst geschrieben und ausgedruckt. Die Wundversorgung von kleineren Wunden, Panaritien und Abszessen wurde mit 5 min/Fall gewertet. Der Zeitaufwand des Pflegepersonals betrug durchschnittlich 18,4 ± 4 min für die Triage, Vorbereitung, Assistenz und Nachbereitung. Die Anlage eines stützenden Verbandes wurde mit durchschnittlich 19,7 ± 3 min dokumentiert. Somit ergeben sich Zeitaufwände von 60.827,4 min (166,7 min; 2,8 h/Tag) für das ärztliche Personal und 78.036,3 min (213,8 min; 3,6 h/Tag) für das Pflegepersonal.

### Kosten-Erlös-Kalkulation

Anhand der Vorstellungstage und Vorstellungsuhrzeiten wurden die behandelten 4088 Patienten den EBM-Ziffern zugeteilt. Es wurden 2109 Patienten (51,6 %) der EBM-Ziffer 01205 zugeordnet (4,79 €/Fall; Grundvergütung von 10.102.11 €). Es wurden 1979 Patienten (48,4 %) der EBM-Ziffer 01207 zugeordnet (8,52 €/Fall; Grundvergütung von 16.861,08 €). Für das Gesamtkollektiv dieser Arbeit ergibt sich somit eine Gesamtgrundvergütung von 26.963,19 €.

Zusammen mit der Abrechnung der Wundversorgungen (EBM 02300; 6,07 €/Patient; 65 Patienten), größerem Aufwand (EBM 02301 „kleinchirurgischer Eingriff II“; 13,74 €/Patient; 30 Patienten) sowie der stabilisierenden Verbände (EBM 02350 „fixierender Verband mit Einschluss mindestens eines großen Gelenkes unter Verwendung unelastischer, individuell anmodellierbarer, nicht weiter verwendbarer Materialien“; 11,29 €/Patient; 143 Patienten) ergab sich somit eine Gesamtvergütung von *29.384,31* *€* für den retrospektiv untersuchten Zeitraum von Januar 2019 bis Dezember 2019 (12 Monate).

Für diese Studie wurde, wie im Abschn. „Studiendesign und Untersuchungsmethoden“ beschrieben, ein Entgelt von 0,62 €/min für das ärztliche sowie 0,42 €/min für das pflegerische Personal berechnet. Im Jahre 2019 betrugen die daraus errechneten Personalbehandlungskosten somit 64.367,30 € (Tab. 2).

**Table 2:** 

*Arzt – Tätigkeit*	*Durchschnittliche Dauer in min*	*Kosten*
Aufnahme + Abschlussgespräch + Erstellung des Arztbriefes	14,8 ± 3	9,18 €
Wundversorgung von kleineren Wunden/Abszessen/Panaritien	5	3,10 €
*Pflege – Tätigkeit*		
Triage, Vorbereitung, Assistenz, Nachbereitung	18,4 ± 4	7,73 €
Anlage stützender Verband	19,7 ± 3	8,27 €

Zusammen mit der Klinikapotheke und dem Einkauf der Universitätsklinik Frankfurt wurden die Kosten für die für die Versorgung von kleinen Wunden, Panaritien und Abszessen benötigten Materialien erhoben. Diese sind in Tab. 3 in Form ihrer jeweiligen Einkaufspreise aufgeführt. Ebenfalls wurden die Kosten für das für die Anlage eines stabilisierenden Verbandes notwendige Material erhoben. Diese sind in Tab. 4 in Form ihrer jeweiligen Einkaufspreise aufgeführt.

**Table 3:** 

Verbrauchsmaterial	Anzahl	Kosten
Wundversorgungsset, steril (Einmalinstrumente)	1	9,30 €
Tupfer, steril (3er-Pack)	1	0,21 €
Abdecktuch, steril	1	0,46 €
Lochtuch, steril	1	0,57 €
Haube	1	0,05 €
Mundschutz	2	0,07 €
Handschuhe, steril	1	0,31 €
Nahtmaterial/Klammerpflaster	1	3,91 €
Einmalskalpell, steril	1	3,50 €
Spritze 10 ml, steril	1	0,04 €
Kanüle, steril	1	0,01 €
Kompressen, steril	1	0,03 €
Mullbinde	1	0,07 €
NaCl, 0,9 %ig, 10 ml	1	0,06 €
Desinfektion (z. B. Octenisept, Braunol©)	150 ml	0,89 €
Lokalanästhetikum (z. B. Mecain)	5 ml	0,15 €
–	*Summe*	*=19,63* *€*

**Table 4:** 

Verbrauchsmaterial	Anzahl	Kosten
Scotchcast® One-Step-Schiene	1	19,31 €
Schlauchverband	1	0,71 €
Verbandwatte	2	1,80 €
Halbelastische Binde	2	1,67 €
–	*Summe*	*=23,49* *€*

Bei 95 Patienten erfolgte eine chirurgische Wundversorgung (65 kleine Wunden, 30 kleine Panaritien/Abszesse). Bei 143 Patienten wurde die Anlage eines stützenden Verbandes notwendig. Insgesamt betrugen die Materialkosten also 5223,92 €. Die Gesamtkosten für die Behandlung wurden somit mit 69.591,22 € berechnet. Das entspricht einem Kostenpunkt von 17,02 €/Patient. Somit wurden für das Jahr 2019 ein Gesamterlös von 29.384,31 € und Gesamtkosten von 69.591,22 € berechnet.

Insgesamt lässt sich ein Erlösdefizit von 40.206,91 € für das Jahr 2019 berechnen. Das entspricht einem monetären Defizit von 9,84 €/Patient.

## Diskussion

Die wichtigste Erkenntnis dieser Studie ist, dass durch die sich eigenständig fußläufig vorstellenden Patienten mit Bagatellverletzungen nichtunerhebliche Kosten entstehen. Für das Universitätsklinikum Frankfurt konnte für das Jahr 2019 beispielhaft insgesamt ein Erlösdefizit von 40.206,91 € errechnet werden, welches allein durch die Behandlung dieser Patienten entstanden ist. Aus dem gesamten Erlösdefizit ergibt sich ein berechnetes finanzielles Defizit von nahezu 10 €/Patient. Bei einem Anteil der Bagatellverletzungen von nahezu 45 % aller unfallchirurgischen Patienten in dieser Studie trägt der Kostenfaktor von rund 40.000 €, welcher allein durch die Behandlung von Bagatellverletzungen in einer einzelnen Notaufnahme entsteht und somit nicht unerheblich zur regelhaften Kostenunterdeckung der Notaufnahmen in Deutschland bei.

Dabei deckt sich der durchschnittliche zeitliche Behandlungsaufwand eines Patienten mit einer Bagatellverletzung von 14,8 min/Patient mit dem ermittelten aus vorangehenden Studien [18]. Obwohl die Behandlung von Bagatellverletzungen für die Berufsgruppen in der Notaufnahme meistens keine große Herausforderung darstellt, wird dies allein durch die reine Masse an täglichen Patienten zum relevanten Arbeitsaufwand. Anhand der kalkulierten Arbeitszeitaufwände von 2,8 h/Tag für das ärztliche Personal und 3,6 h/Tag für das Pflegepersonal wird dies verdeutlicht.

## Vorstellungszeiten

Aktuelle Literatur belegt ein Ansteigen der ambulanten Notfälle am Wochenende um etwa 39,0 %. Werktags erfolgen gehäufte ambulante Notfallvorstellungen zwischen 18 und 20 Uhr und am Wochenende zwischen 10 und 12 Uhr [20]. Ebenso belegt dies eine vorangehende Studie des Autors dieser Arbeit aus dem Jahre 2021, bei der ein gehäuftes Patientenaufkommen mit Kopfplatzwunden außerhalb der regulären Öffnungszeiten der niedergelassenen Ärzte aufgezeigt werden konnte [17]. Daten aus dieser Studie zeigten ein balanciertes Aufkommen der ambulanten Notfallversorgung von Bagatellverletzungen innerhalb und außerhalb der regulären Arbeitszeit von Montag bis Freitag zwischen 7:00 Uhr und 17:00 Uhr. Es konnten 2109 Patienten (51,6 %) identifiziert werden, welche sich von Montag bis Freitag zwischen 07:00 und 19:00 Uhr vorstellten.

## Gründe für die hohe Selbstvorstellungsquote

Gründe für die steigenden Vorstellungszahlen in Deutschlands Notaufnahmen [1] könnten der sozioökonomische und demografische Wandel, die gesellschaftliche Beschleunigung und mangelndes Wissen über die gegebenen Strukturen, aber auch veränderte Versorgungspräferenzen und individuelle Patientenpräferenzen darstellen. Viele Patienten fühlen sich in der Notaufnahme besser versorgt. Ebenfalls fürchten manche Patienten lange Wartezeiten beim ärztlichen Bereitschaftsdienst, während dieser einem Großteil der Patienten insgesamt unbekannt zu sein scheint [4, 5, 19]. Allgemein wird deutlich, dass der Bereitschaftsdienst häufig ungesteuert und oft auch nur mit geringfügigen Beschwerden in Anspruch genommen wird, während akute lebensbedrohliche Gesundheitsprobleme eher selten vorkommen [6, 12].

In einer Untersuchung aus dem *Deutsches Ärzteblatt* von 2017 mit über 1000 befragten Patienten in der Notaufnahme, gaben nur 25 % an, mit einer Einweisung durch den Hausarzt oder den Fachspezialisten vorstellig zu werden. Zusätzlich wurde mit abnehmender Schwere und Dringlichkeit des ursächlichen Problems, eine signifikante Abnahme des Anteils an Patienten, welche sich mit Einweisungen durch die genannten Akteure vorstellten, beobachtet. Der deutlich größere Anteil der Patienten besucht die Notaufnahme rein aus eigener Motivation oder auf Empfehlung von Dritten wie Freunden oder Bekannten [19, 22]. Diese Zahlen decken sich daher mit der dokumentierten Selbstvorstellungsquote von 96 % bei Patienten mit lediglich Bagatellverletzungen aus dieser Studie. Mit abnehmender Dringlichkeit und Schwere der Behandlung zeigt sich offenbar sogar eine steigende Tendenz zur eigenverantwortlichen Vorstellung in der Notaufnahme.

Nicht nur die Anzahl von Patienten mit geringfügigen Beschwerden, sondern auch das Anspruchsdenken der Patienten im Rahmen der gesetzlichen Versicherung-Flatrate trägt zu einer erhöhten Arbeitsbelastung von Ärzten in der Notfallversorgung bei [3, 25]. Die Frustrationsschwelle aufseiten der Patienten scheint aufgrund der langen Wartezeiten in den Notfallambulanzen zu sinken. Vorfälle von Gewalt und Provokationen des medizinischen Personals werden zunehmend häufiger dokumentiert [8, 29]. Den Patienten fehlt nicht nur das Bewusstsein für die Dringlichkeit ihrer Verletzung, sondern auch die Kenntnis über die bestehenden Strukturen und den Aufbau der Notfallversorgung unseres Gesundheitssystems für die Notfallversorgung [19, 22]. Patienten sollten genau dorthin gehen, wo ihnen am effizientesten geholfen werden kann. Ebenfalls sollen Ressourcen nur dort verbraucht werden, wo es unbedingt sein muss. Dies ist Teil aktueller Reformpolitik [2].

Auf der Seite des Gesundheitssystems und der Krankenkassen spielen hingegen der begrenzte Zugang zu ambulanter Versorgung und der Bekanntheitsgrad ambulanter Notdienste eine Rolle [22]. Ebenso stellten Somasundram et al. fest, dass Patienten, welche zuvor den ärztlichen Notdienst kontaktierten, in den meisten Fällen telefonisch direkt in die Notaufnahme weiterverwiesen werden und nur nahezu 3 % vom ÄBD untersucht werden [4, 22]. Zusätzlich arbeiten viele Bereitschaftsdienstzentralen heutzutage monetär defizitär, was als ein großes Problem angesehen wird [30]. Da das Patientenaufkommen stetig weitersteigt, wäre zwingend eine bessere Vergütung der Behandlung der Bagatellverletzungen notwendig, um der hier dargelegten Kostenunterdeckung entgegenzuwirken. Aufgrund der steigenden Patientenzahlen mit hohem Anteil an Bagatellverletzungen, und des hier dargestellten hohen zeitlichen Aufwands für ihre Behandlung, müssen zumindest die Kosten der Behandlung aufseiten der Klinik gedeckt werden.

## Limitationen

Diese Arbeit weist einige Limitationen auf. Obwohl die Berechnung der Kosten für die Behandlung von Patienten mit Bagatellverletzungen den tatsächlichen Kosten nahekommt, bleibt die Berechnung lediglich eine Hochrechnung. Die Kosten wurden näherungsweise erhoben und verbleiben daher nicht exakt. Eine weitere Limitation stellt die Berechnung der Werte anhand des Beispielsjahrs 2019 dar. Jedoch wurde in dieser Arbeit eine hohe Anzahl an Patienten untersucht. Ein signifikanter Unterschied der durchschnittlichen Behandlungsdauer und -kosten zwischen unterschiedlichen Jahrgängen bleibt daher unwahrscheinlich. Jedoch sind so keine Aussagen zu den durchschnittlichen Kosten und Erlösen innerhalb eines Jahres möglich.

## Fazit für die Praxis

Schlussendlich wird klar: Es herrscht eine zu große Knappheit an der medizinischen Ressource „Personal“, um das heutzutage hohe Aufkommen an sich selbst vorstellenden fußläufigen Patienten mit Bagatellverletzungen zufriedenstellend und ökonomisch zu bewältigen. Die bisherige Vergütung der Behandlung von Bagatellverletzungen durch den einheitlichen Bewertungsmaßstab ist für den Krankenhaussektor höchst unzureichend.
